# Biodegradable and Non-Biodegradable Biomaterials and Their Effect on Cell Differentiation

**DOI:** 10.3390/ijms232416185

**Published:** 2022-12-19

**Authors:** Rency Geevarghese, Seyedeh Sara Sajjadi, Andrzej Hudecki, Samad Sajjadi, Nahid Rezvani Jalal, Tayyebeh Madrakian, Mazaher Ahmadi, Małgorzata K. Włodarczyk-Biegun, Saeid Ghavami, Wirginia Likus, Krzysztof Siemianowicz, Marek J. Łos

**Affiliations:** 1 Biotechnology Center, Silesian University of Technology, 44-100 Gliwice, Poland; 2 School of Allied Medical Sciences, Shahid Beheshti University of Medical Sciences, Tehran 1971653313, Iran; 3 Łukasiewicz Network-Institute of Non-Ferrous Metals, 44-121 Gliwice, Poland; 4 Faculty of Chemistry, Bu-Ali Sina University, Hamedan 6516738695, Iran; 5 Autophagy Research Center, Shiraz University of Medical Sciences, Shiraz 7134845794, Iran; 6 Polymer Science, Zernike Institute for Advanced Materials, University of Groningen, Nijenborgh 4, 9747 AG Groningen, The Netherlands; 7 Department of Human Anatomy and Cell Science, University of Manitoba College of Medicine, Winnipeg, MB R3E 0V9, Canada; 8 Research Institutes of Oncology and Hematology, Cancer Care Manitoba-University of Manitoba, Winnipeg, MB R3E 0V9, Canada; 9 Biology of Breathing Theme, Children Hospital Research Institute of Manitoba, University of Manitoba, Winnipeg, MB R3E 0V9, Canada; 10 Faculty of Medicine in Zabrze, University of Technology in Katowice, 41-800 Zabrze, Poland; 11 Department of Anatomy, Faculty of Health Sciences in Katowice, Medical University of Silesia, 40-752 Katowice, Poland; 12 Department of Biochemistry, Faculty of Medicine in Katowice, Medical University of Silesia, 40-752 Katowice, Poland

**Keywords:** biological cues, collagen, gelatin, lactic-co-glycolic acid, L-lactic acid, matrigel, polycaprolactone, polyethylene glycol, polyethylene terephthalate, transdifferentiation

## Abstract

Biomaterials for tissue scaffolds are key components in modern tissue engineering and regenerative medicine. Targeted reconstructive therapies require a proper choice of biomaterial and an adequate choice of cells to be seeded on it. The introduction of stem cells, and the transdifferentiation procedures, into regenerative medicine opened a new era and created new challenges for modern biomaterials. They must not only fulfill the mechanical functions of a scaffold for implanted cells and represent the expected mechanical strength of the artificial tissue, but furthermore, they should also assure their survival and, if possible, affect their desired way of differentiation. This paper aims to review how modern biomaterials, including synthetic (i.e., polylactic acid, polyurethane, polyvinyl alcohol, polyethylene terephthalate, ceramics) and natural (i.e., silk fibroin, decellularized scaffolds), both non-biodegradable and biodegradable, could influence (tissue) stem cells fate, regulate and direct their differentiation into desired target somatic cells.

## 1. Introduction

**Scheme 1:**
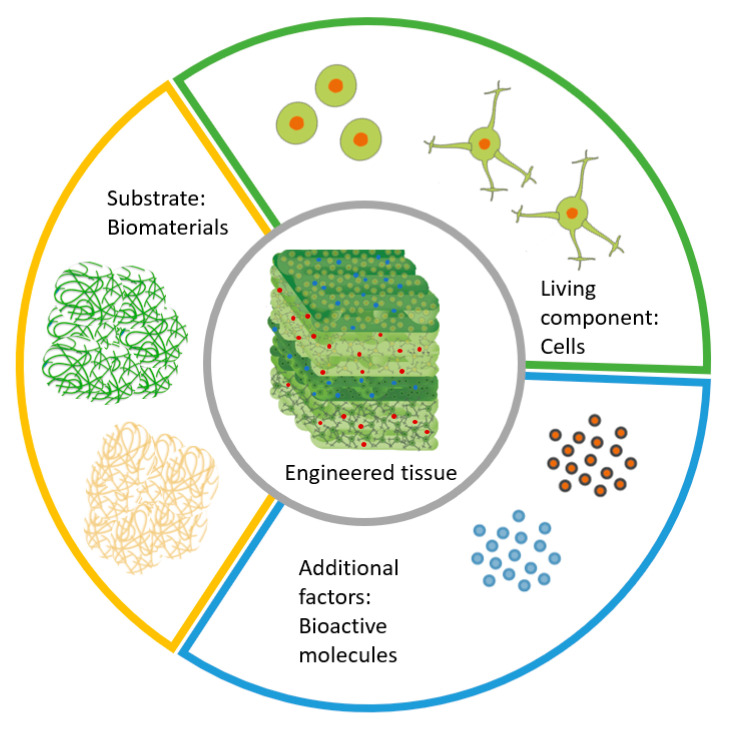
Tissue engineering of cells, signals, and the biomaterials scaffold, which acts as a template for tissue formation by allowing cells to migrate, adhere, and produce tissue. The signal could be provided chemically by growth factors or physically by a bioreactor. Cells used for a scaffold settlement could be somatic cells, adult stem cells of various origins, embryonic-derived stem cells, cells obtained by transdifferentiation, or induced pluripotent stem cells. Materials used for a scaffold can be either non-biodegradable or biodegradable. The latter enables a neo-tissue formation with a new extracellular matrix replacing a degraded biomaterial.

The impaired function of organs, tissues, and cells due to damage or defects generate unmet needs for autologous transplants. The rapid development of regenerative medicine offers the opportunity to develop artificial tissue transplants, often based on engineered biomaterials, to replace the lost, natural tissue scaffolds. Tissue engineering is a fast-emerging science that aims to develop tools for the regeneration of damaged or diseased tissues and organs [1,2]. Modern tissue replacement is based on two pillars, biomaterials for tissue scaffolds and utilization of (preferably autologous) human cells as a therapeutic agent [3]. An artificial tissue could be created by seeding cells in a proper microenvironment delivered by carefully engineered biomaterial scaffolds that deliver appropriate biochemicals and biophysical cues [4]. The tissue engineering triad includes cells, signals, and the scaffold, which acts as a template for tissue formation by allowing cells to migrate, adhere, and produce tissue (Scheme 1) [5]. Per definition, a biomaterial is a substance that has been created to interact with biological systems for therapeutic or diagnostic purposes. Biomaterials can be produced from natural materials or created in the laboratory using several chemical methods [6].

Cells used for a scaffold settlement could be somatic cells, adult stem cells of various origins [7,8,9], embryonic-derived stem cells, cells obtained by transdifferentiation, or induced pluripotent stem cells [10,11,12] (Scheme 1). Different kinds of stem cells are used to cure abnormalities and increase tissue repair and regeneration for various cell types [13]. Cyclooxygenase 2 upstream of the IL-4 gene, B cells (NF-B), IL-4 as a regulator of macrophages from promoter gene, and multiple consensus elements for the nuclear factor kappa-light-chain have been used for gene editing (i.e., inflammation, homing, and retention, amplification and increased expression of anti-cytokine drugs such IL-1Ra in response to IL-1, improving responses to inflammatory cytokines [14,15,16]. Mesenchymal stem cells (MSCs) from bone marrow have been used for mandibular, metatarsal, femoral head, femurs, tibial, tibial diaphyseal defect, craniofacial, inferior orbital rim bone, and jaw bone loss in bone marrow tissue engineering [17,18,19,20]. MSCs have also been used for the umbilical cord and skin tissue engineering (from umbilical cord blood and bone marrow, respectively) for the treatment of radial defeat and mending burn wounds, healing, keratinization, and increased vascularization [21,22]. Skeletal muscle myeloid-derived suppressor cells (MDSCs) have been employed to treat skull and calvarial defeats/diseases [20]. MDSCs derived from the orbicular oris muscle have been used to treat cranial defects [23]. Adipose-derived stromal cells (ASCs) and MSCs from adipose tissue have been used in the treatment of parietal bones, ulna, Osteoarthitis OA-like damage, and jaw bone for adipose tissue engineering [24,25,26]. Free cell transplantation results in only about 10% of cells engrafted at the targeted site, while even this value varies broadly depending on cell type, implantation method, and implantation site. Biomaterials also have other goals, to encourage cell engraftment and reduce cell loss. Biomaterials should not only aid in cell-cell interactions but also help in the deposition of native ECM and support cell survival [4]. The usage of different stem cells opens a new era in regenerative medicine and creates new challenges. Phenotypic changes during embryonic development are a good example of extracellular matrix (ECM)-cell interactions necessary for proper stem cell differentiation [27].

Materials used for a scaffold can be either non-biodegradable or biodegradable. The latter enables a neo-tissue formation with a new ECM replacing a degraded biomaterial. Considering the tissue response to the implantation, classical biomaterials can be divided into biotolerant, bioactive, and bioinert [28]. Modern biomaterials should be engineered for individual patients considering stem cells intended to be settled on the scaffold and their desired way of differentiation. It creates a new area for research and bioengineering, giving a new meaning to the term “bioactive scaffold”.

## 2. Natural Biomaterials Directing Differentiation in Desired Directions

Natural biomaterials are biodegradable and biocompatible entities derived from plant and animal sources [29,30,31]. Proteins (e.g., collagen, fibrin, elastin, silk) and/or polysaccharides (e.g., cellulose, dextran, chitosan, and glycosaminoglycans) and plant-derived biomorphic carbon material, mainly constitute natural biomaterials. The ECM serves as a crucial component in the stem cell microenvironment [32]. ECM components, besides serving as a mechanical support for cell adhesion, are composed of bioactive compounds which regulate cell growth and differentiation through direct binding with specific cell surface integrin or non-canonical growth factor presentation [33,34]. Natural biomaterials could mimic the ECM composition and contain intrinsic biological cues, which provide favorable environments for tissue engineering applications [35,36]. Besides biochemical properties, ECM physical characteristics, i.e., stiffness, absorbency, and topography, are capable of influencing stem cell differentiation [37].

**Figure 1:**
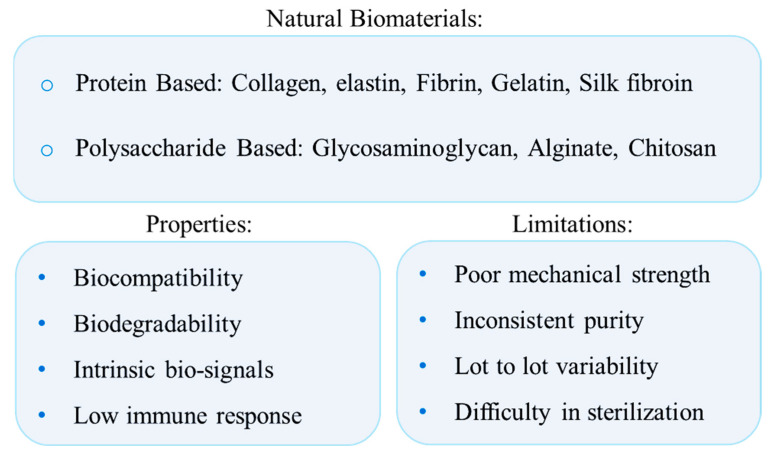
Natural biomaterials. Please, see the main text above for detailed information.

Although natural biomaterials are being widely used, inconsistent purity arising from lot-to-lot variability and difficulty in sterilization and purification is usually the main limitations of natural biomaterials. It should be noted that all these features may not be present in all natural biomaterials (Figure 1) [35,38].

### 2.1. Collagen

One of the earliest natural biomaterials to be identified and isolated is collagen [31]. This protein is the main constituent of the ECM and can be formed into three-dimensional (3D) scaffolds with the potential to influence cell growth, morphology, and function [31,39]. Outstanding characteristics, e.g., (partial) self-assembly under physiological conditions, biocompatibility, degradability, mechanical strength, and adhering properties, lead collagen to be one of the most utilized components with versatile usage for tissue engineering purposes [31,40]. For instance, collagen is employed for skin restoration experiments due to its high rate of degradation/replacement by endogenous components of ECM [40]. Also, owing to its potential to promote cell adhesion and migration, collagen has been applied in tissue repair models [31]. Collagen matrices were also shown to serve as a support for epithelial cell growth and differentiation in diabetic wounds [41]. Various collagen-based scaffolds have been utilized for cell differentiation purposes. Such scaffolds play a great role in regulating MSC differentiation into desired cell types [31,41,42]. Type I collagen induces signals to promote cell proliferation via the MAPK/ERK pathway [43]. This could be affected by focal adhesion complexes, which contain integrin, vinculin, paxillin, and other proteins that form thick bundles of collagen fibrils [43].

Collagen could be combined with natural and/or synthetic polymers to improvise its bio-mimicking properties. In this context, collagen-glycosaminoglycan (CG) scaffolds are utilized as a stable culture of MSCs to induce differentiation toward tendon, cartilage, and osteogenic phenotypes [44,45]. Moreover, Ryan et al. have introduced a collagen-based scaffold, functionalized with copper-eluting bioactive glass, that could serve as a treatment for osteomyelitis. This model incorporates the controlled release of non-antibiotic antibacterial to reduce infection and enhance osteogenesis and angiogenesis both in vitro and in vivo [46].

#### Topology and Stiffness

There are 29 identified collagen subtypes, among which the five most prevalent ones found in the human body are types I, II, III, IV, and IX [47]. Triple helical structures are present in all collagen subtypes. Collagen fibers are made up of tightly packed collagen fibrils that are 30–100 nm thick and 1–20 m long [47].

To increase scaffold rigidity, chemical modifications of the ECM are mainly achieved through collagen glycation or crosslinking techniques. However, such ways induce minor increases in ECM stiffnesses and may result in undesired effects, for instance, prolonged incubations. Hence, the introduction of new bioactive ligands and/or alterations to the ECM architecture is of interest [48]. A study showed that the stiffness of 3D ECM could play a great role in cell migration behavior by controlling cell volume homeostasis. For this, three types of collagen-based hydrogels with tunable stiffness, including collagen, collagen-alginate with 11 mg/mL CaCl_2_, and collagen-alginate hydrogel with 56 mg/mL CaCl_2,_ were utilized to embed the MDA-MB-231 cells. The results showed that the cell volume homeostasis and migration speed of the cultured cells is controlled by ECM rigidity [49]. Also, the results of a study on the role of 3D alginate/collagen-I interpenetrating networks stiffness on fibroblast biology suggested that the adjustment of a dressing biomaterial stiffness that is placed on a wound site could be considered an applicable approach for skin repair and regeneration [50]. Since the collagen gel itself is fragile, generally, collagen-based biomaterials are reinforced through chemical or physical crosslinking techniques to induce differentiation into hard tissues. Takitoh et al. showed that the osteogenic activity of MSCs was higher on the gamma-cross-linked nonfibrillar collagen gels compared to the non-irradiated fibrillar substrates. Due to mechanical signal transduction, the formation of the focal adhesion was lower on the gamma-cross-linked nonfibrillar gels. Thus proteins with regulatory functions might be absorbed more efficiently, resulting in the promotion of MSCs differentiation into osteoblasts [33].

### 2.2. Elastin

Elastin is a key component of the ECM with intriguing qualities, including biocompatibility, biodegradability, and elasticity. Elastin’s elasticity, which is its most striking mechanical characteristic, has made it a material of interest for creating scaffolds (i.e., vascular grafts and skin substitutes). High porosity hydrogels comprised of elastin or polymers that resemble it are being utilized as 3D cell cultures, drug delivery, and gene delivery systems. However, elastin is not utilized as frequently as other proteins in the synthesis of hydrogels since its purification is complicated. Moreover, elastin has the propensity to calcify [51]. Various forms of naturally occurring (i.e., decellularized tissue, insoluble elastin, tropoelastin, hydrolyzed elastin) and biosynthetic elastin (i.e., tropoelastin, elastin-like polypeptide, and hybrids with other molecules) with different molecular mass exist [52]. Depending on the utility, different methods can be employed to create elastin-based hydrogels, including electrospinning, self-assembly crosslinking, and glutaraldehyde as a crosslinking agent [51].

#### Topology and Stiffness

In terms of structure, elastic fibers primarily consist of two parts: an inner core of amorphous crosslinked elastin and an exterior microfibrillar mantle (microfibrils) which are 10–12 nm in diameter and primarily composed of fibrillin-1 [51,52].

The mechanical properties of elastin and the hydrogel’s porosity can be changed during the synthesis process. For instance, to increase the pore size for a highly porous hydrogel, high-pressure CO_2_ injection at controlled pressure and temperature during the fabrication is a useful strategy. To increase the elasticity of natural hydrogels, elastin is often utilized [51,53]. Elastin by itself does not provide rigidity to hydrogels. Hence for promoting stiffness and structural support, the inclusion of a chemical crosslinking that produces covalent bonds is beneficial [53]. For instance, collagen/elastin hydrogels crosslinked by squaric acid were shown to be stiffer and more resistant to enzymatic degradation than those that are unmodified [54].

### 2.3. Fibrin

Fibrin is derived from fibrinogen. Fibrin plays a key role in the coagulation cascade and natural tissue healing process. Fibrin helps to promote cell differentiation, proliferation, function, and survival by attaching to cell surface receptors like integrins and serving as a sturdy 3D scaffold [55]. Other properties include biocompatibility, rapid biodegradability, and easy fabrication. Because they can be made simply from patient blood, these gels are viewed as an alternative to collagen [55,56]. 

#### Topology and Stiffness

Fibrin-based hydrogels are preferred in cardiac tissue engineering. However, due to their mechanical weakness, hydrogels might eventually fail in the dynamic stressful environment of the heart. Therefore, a cardiac patch’s ability to mechanically and functionally integrate with the native myocardium is crucial. For a variety of tissue engineering applications, microthreads generated from natural biopolymers (e.g., fibrin) that control cellular orientation and have tunable mechanical properties have been studied. As such, Chrobak et al. developed a model of composite layers with tunable, mechanical patch properties that could facilitate cell alignment and support cell functionality [57].

### 2.4. Gelatin

Gelatin, a derivative of collagen, is formed into biomaterial through heat and enzymatic degradation [31,58]. Gelatin has common molecular composition and characteristics with collagen such as biocompatibility, and biodegradability. Certain features such as lower cost compared to other ECM proteins, high solubility, molecular composition similarity to collagen, low rate of antigenicity, and cell toxicity make gelatin a suitable biomaterial for tissue engineering purposes [59]. However, it should be considered that for long-term objectives such as cell differentiation and wound healing experiments, gelatin is not long-lasting enough, as it degrades quite fast. Moreover, gelatin is highly susceptible to several proteases [59,60]. Yet, the recent advancement of manufacturing technology is fading such drawbacks. For example, gelatin composites have improved properties such as mechanical strength, biocompatibility, and bioactivity (proliferation, differentiation) [59]. In one study, Tajima et al. showed that the incorporation of gelatin hydrogel microspheres improved the culture condition (i.e., improved oxygen and nutrients permeation into the cell aggregates for longer periods), leading to improved survival, proliferation, and osteogenic differentiation [61]. Furthermore, gelatin and gelatin composites can be utilized in producing microparticles for cell and tissue cultures. Cytokines can be loaded on gelatin microparticles for the directed differentiation of cells [59]. Cruz et al. utilized gelatin microparticles loaded with TGF-β1 to induce chondrogenesis to bone marrow-derived cell spheroids [62].

#### Topology and Stiffness

The protein composition of gelatin is similar to collagen. However, it cannot form triple helices and, subsequently, fibrillar networks of in vivo tissues that are present in collagen. This limitation is considerable as the structure plays pivotal functions in directing cell behavior. Gelatin-methacrylate (gelMA) is utilized as effective ECM-based matrices. GelMA preserves constant gelatin concentration and offers many physical properties. Berger et al. proposed a method to decouple fiber density and scaffold stiffness by constructing an interpenetrating network hydrogel of gelMA and collagen type I. While this method retains the fibrillar structure of collagen, it permits the formation of a wide range of shear moduli. Moreover, by adjusting the gelMA to collagen ratio, this method can alter matrix fiber density without affecting the amount of protein [48]. As mentioned, composite microparticles overcome the limitations of gelatin and other types of biomaterial. Kozlowska et al. have shown that collagen-gelatin composite microparticles result in advanced mechanical stability and higher resistance to dissolution than pure collagen or gelatin [63].

### 2.5. Silk Fibroin

Silk fibers are composed of a filament core protein named fibroin and a coating comprising sericin proteins, an outstanding natural protein for 3D scaffold and biomaterial coating applications owing to their strong hydrogen bonding and highly durable capacity [64]. Silk fibroin is utilized for both in vitro and in vivo purposes, respectively, to support stem cell adhesion, proliferation, and differentiation and to promote tissue repair [65]. Silk scaffolds’ immunogenicity and antigenicity are well tested, and results show that they are generally well tolerated [66]. The optimal biocompatibility of silk products can be compared with other biomaterials, such as poly (lactic acid) and collagen [66]. Silk fibroin scaffolds are well adapted to mild manufacturing conditions compatible with growth factor-loaded scaffold production (e.g., temperature, organic solvents, pH, during processing) [67]. Silk systems retain strength for a long time which is in favor where slow degradation and load-bearing capacity are required [66].

#### Topology and Stiffness

A heavy (390 kDa) and a light (26 KDa) chain linked by disulfide bonds consist of the main structure of silk fibroins. These chains are hydrophobically linked to a P25 (25 KDa) glycoprotein with a molar ratio of 6:6:1, respectively [66,68]. Silk fibroins fibers are characterized by excellent mechanical properties, i.e., large break strain (4–26%), great strength (300–740 MPa), and outstanding toughness (70–78 MJ m^−3^) that is even greater than several synthetic fibers, and some collagens such as tendon collagen (7.5 MJ m^−3^). Owing to such mechanical properties, silk fibroin is preferred for load-bearing tissue engineering applications. In biomaterial engineering silk fibroin scaffolds are often made from regenerated silk fibroin solutions lacking hierarchical and secondary structures which results in the production of delicate and weak scaffolds. To overcome this, several strategies (e.g., crosslinking, porogens, and 3D bioprinting) have been proposed [68]. 

### 2.6. Glycosaminoglycans

GAGs refer to six major polysaccharides, including hyaluronic acid (HA), chondroitin sulfate (CS), dermatan sulfate (DS), keratan sulfate (KS), and heparan sulfate (HS) and heparin (HP) [69]. GAGs highly recapitulate the ECM in tissue-engineered constructs allowing them to be vastly utilized in biomedical sciences for promoting stem cell differentiation or phenotypic maintenance of transplanted cells. These properties direct its application in wound healing tissue engineering constructs (i.e., skin and cornea), restoring damaged tissue (i.e., cartilage, bone), and neuronal regeneration [51].

#### Topology and Stiffness

Glycosaminoglycans (GAGs) are long, unbranched polysaccharide chains mainly composed of repeating disaccharide units linked by glycosidic bonds [70].The composition of these units differentiates GAGs and the molecular mass which ranges from a few kDa to several million Da for hyaluronan [69]. The inclusion of GAGs within tissue-engineered scaffolds could aid their assembly. Hydrogels containing linked heparin-hyaluronan molecules show greater stiffness [71]. The stiffness of HA-based hydrogels could be modified in several kPa without affecting adhesion strength through varying crosslinking, cross-linker concentration, or photoinitiator concentration. This stiffness tunability allows the hydrogel system to control the development of human liver stem cells, MSCs, and neural progenitor cells [72].

### 2.7. Alginate and Chitosan

Alginate is a naturally-occurring, algae-derived polysaccharide that is broadly utilized for tissue engineering and regeneration because of its specific characteristics such as biocompatibility, non-thrombogenic nature, affordability and structural similarity to the ECM [73]. Alginate hydrogels are used as biomimetic matrices, drug transporters, and substrates for cell encapsulation and transplantation for different cell populations intended to direct the regeneration and function restoration of tissues and organs [74]. As an example, MSC is primarily delivered intramyocardially using alginate hydrogel to improve cell retention and cell-mediated cardiac healing [73]. Alginate hydrogels also have been used in tissue engineering to regenerate bone, cartilage, and liver [74]. Chitosan is a natural polymer commonly derived from crustacean shells. Chitosan is well known for hydrophilicity, biocompatibility, and biodegradability. The cationic character of chitosan permits the formation of polyelectrolyte complexes (PEC) with anionic polymers such as alginate [75]. This complex offers the benefits of each polymer while constraining its disadvantages. Alginate, as an anionic polymer, could form PECs with chitosan through electrostatic interactions. 3D porous Chitosan-alginate (CA) scaffolds can promote the proliferation and enrichment of cancer stem-like cells [75].

#### Topology and Stiffness

CA-based hydrogels in a polyacrylamide-crosslinked network can improve mechanical properties and biodegradability. This hydrogel has a highly interconnected porous structure and ladder-like fibrous topology, which facilitates bone osteoblast cell attachment and proliferation and biomineralization [76]. 3D porous CA scaffold stiffness could promote different responses. It was shown in prostate cancer cell lines where the CA culture platform with various stiffness supported prostate cancer growth and phenotypic expression. In detail, three compositions of 3D porous CA scaffolds (2, 4, and 6 wt%) with PC-3, C4-2B, and 22Rv1 cell lines were utilized to assess the effect of scaffold stiffness. Among the cell lines, the PC-3 formed clusters, while the other two formed multicellular spheroids. Moreover, unlike PC-3, the other two lines were mineralized in basal media. This showed that CA scaffold cultures exposed differences in PCa phenotypes [75]. CA scaffolds lack integrin-binding ligands, resulting in rounded cell morphology and limited cell-substance interactions. In one study, CA scaffolds were fabricated with 2, 4, and 6 wt% (mimicking the normal breast tissue, primary breast cancer, and bone metastases stiffness) while the breast cancer cell line MDA-MB-231-GFP was cultured on these compositions to assess proliferation, morphology, and migration in response to scaffold stiffness. Cells cultured with 6 wt% CA had the highest migration rate, followed by 4 wt% CA, 2D culture, and 2 wt% CA. These results suggest that 231 cells recognized the stiffness of CA scaffolds despite the absence of focal adhesions, suggesting that non-integrin-based mechanisms can explain the observed mechanotransduction responses [77].

### 2.8. Other Natural Biomaterials

#### 2.8.1. Plant-Derived Biomorphous Carbon Materials

Plant-derived biomorphic carbon materials derived by pyrolysis exhibit highly-porous structures at the macro- and micro-level, as well as biocompatibility. They attracted marked interest as potential scaffolds for bone substitutes from several researchers, including our group. The structure and properties of such materials are mainly influenced by the plant precursors used and pyrolysis temperature [78,79,80,81]. The Finnish group has used raw as well as annealed at 140 °C and 200 °C monolithic shapes of the birch (*Betula pubescens*). Their studies have shown that wood implanted into rabbit bone had been involved in the bone regeneration process. Heat-treated wood can behave as a porous biomaterial scaffold, allowing the growth and differentiation of host bone and cartilage as small islets into the wood implants, presenting osteoconductive contact and attachment at the interface. The annealing temperatures had a positive impact on this phenomenon as well as on the liquid penetrability (e.g., blood).

#### 2.8.2. Matrigel

Matrigel, a basement membrane matrix extracted from mouse sarcomas, has extensively been investigated for cell cultures and stem cell differentiation [82]. Matrigel is composed of four major ECM proteins, i.e., laminin, collagen IV, entactin, and heparin sulfate proteoglycan, and also contains tumor-derived proteins, including growth factors as well as enzymes [83]. Hence, cells seeded in matrigel-based substrates benefit not only from biochemical cues but also from the mechanical arrangement of their components [84]. For example, matrigel may alter adipocyte yield and lipogenesis by inducing preadipocyte differentiation to mature adipocytes [82]. It should be noted that matrigel is not recommended for clinical use as this substrate may stimulate teratoma development [84]. Kaiser et al. showed that human neural progenitor cell lines seeded on matrigel survived better than their counterparts planted on surfaces that were not coated with matrigel. Furthermore, cells seeded on matrigel showed strong synaptic marker signatures and differentiated into neuronal cells to a greater extent. Probably the above effect is at least in part because matrigel provides both mechanical and trophic supports [84].

### 2.9. Decellularized Scaffolds as an Example of Natural Biomaterials with Differentiation-Supporting Properties

A promising technique for the preparation of a natural biomatrix scaffold is organ decellularization [85]. Decellularized ECM (dECM) is one of the most valued natural biomaterials due to its renowned features, i.e., complex composition, vascular networks, and unique 3D structure that mimics the native complexed physical and chemical profile [52,86]. In such scaffolds, despite decellularization, ECM can retain tissue-specific components, including proteins, growth factors, and nanovesicles. Tissue or organ decellularization is obtained by removing cells while maintaining the structural design of ECM and cellular niche through physical, chemical, and biological methods [51,87]. These naturally derived ECMs could be used as patches, powders, and hydrogels [88]. Such constructs could degrade slowly and be replaced by the host ECM proteins [89]. dECM derived from various organs can serve as biological support to guide cell adhesion, migration, growth, and differentiation [90]. Biochemical cues, provided by decellularized hydrogels and dECM, are of key importance for tissue function, e.g., preserving stem cells’ stemness property and directing stem cell differentiation toward specific lineages [86,91]. For example, kidney dECM maintains biochemical and biophysical cues which regulate cell differentiation [92]. Hence, in a study renal ECM scaffolds are capable of inducing embryonic stem cell differentiation toward meso-endodermal lineage [93]. Other examples of decellularized scaffolds’ capability to induce differentiation are namely: scaffolds derived from human decellularized adipose tissue to support hematopoietic progenitor cell differentiation towards the pro-angiogenic monocyte/macrophage lineage [94]; bladder scaffolds capable of inducing blastema cells differentiation into epithelial and fibroblast cells [95]; rat acellular liver scaffold models promoting human liver stem cells differentiation into functional hepatocytes, as well as epithelial and endothelial-like cells [96]; myocardial-, and vessel-derived dECM supporting survival and differentiation of cardiac progenitor cells into cardiovascular lineage cells [97].

The decellularized scaffolds are superior to tissue culture plastics or natural biomaterials for guiding stem cell differentiation, probably due to their multifunctional-3D structure that provides necessary signals to modulate cell function [86]. For instance, hepatocyte-like cells differentiated from adipose-derived stem cells have a higher degree of phenotypic and functional similarity to primary hepatocytes when obtained through dECM compared to cultures on type I collagen matrix [98]. Also, decellularized MSC derived from mouse bone marrow enhanced adipogenic and osteogenic differentiation when compared to plastic, fibronectin, and type I collagen-coated plates [99].

dECM has been considered as promising cell culture substrates with differentiation properties to the desired lineage of the reseeded stem cells. Decellularized scaffolds serve as a guide for the deposited cells. Accordingly, dECM deposited by uninduced MSCs enhanced stemness properties, while dECM placed by osteogenic MSCs induced differentiation into osteoblasts [100]. When comparing tissue-specific (e.g., neural stem cells) to non-tissue-specific (e.g., human placenta) decellularized hydrogels, the specific ones have the potential to guide stem cell differentiation towards the appropriate lineage, probably due to certain ECM properties, e.g., matrix stiffness, organization, and biochemistry [88]. Accordingly, French et al. showed that cardiac progenitor cells seeded in naturally-derived decellularized cardiac ECM had enhanced differentiation toward the cardiac lineage and decreased maturation toward the fibroblastic lineage in comparison with collagen scaffolds, highlighting the significance of tissue-specific ECM cues that regulate progenitor cell behavior [101]. Moreover, Viswanath et al. assessed the feasibility of regenerating the spinal cord from apical papilla-derived MSC within 3 different hydrogels characterized by distinct structural, mechanical, and biological properties (i.e., bone, spinal cord, and dentine-derived dECM hydrogels). Accordingly, although all hydrogels supported cell viability and proliferation, spinal cord and bone-derived hydrogels enhanced neural lineage markers expression. The findings confirmed that tissue-specific ECM scaffolds significantly affected the progenitor behavior; hence apical papilla stem cells’ differentiation to a neural lineage was more evident within those seeded in spinal cord scaffolds compared to the other biomaterials [102].

On the other hand, as a limitation, it should be noted that decellularized ECM tissue-specific architecture often constraints their application to reduced options (i.e., the specific tissue that they have been derived from). For instance, decellularized heart valves and vasculature applications are restricted to heart valve replacement and vascular grafts [52]. Also, availability, poor reproducibility, and large batch-to-batch variability are further drawbacks of dECM [103]. Moreover, tissue decellularization is challenged by its limited potential for recellularization. However, this could be addressed by transforming decellularized organs and tissues into hydrogels. This approach enables cells to be encapsulated throughout their structure while retaining tissue-specific cues [87,104].

## 3. Synthetic Biomaterials or Dopants Supporting Differentiation of Stem Cells into Selected Cell TYPES

### 3.1. Synthetic Biomaterials 

Synthetic biomaterials, non-biological in origin, marked their position among the materials for tissue engineering and regenerative medicine, as they are relatively easy to manufacture at a large scale, with high flexibility and control over their composition, microstructure, degradation rate, mechanical properties, and possible functionalization. The most commonly used synthetic biomaterials include polycaprolactone (PCL), poly L-lactic acid (PLLA), poly(ethylene glycol)diacrylate (PEGDA), poly lactic-co-glycolic acid (PLGA), polytetrahydrofuran (PTHF), polyvinyl alcohol (PVA) and polyurethane (PU). Here, we will discuss the strategies employed to guide stem cell differentiation using those materials with a focus on material composition, topology, stiffness, and biological cues associated with the material.

#### 3.1.1. Polycaprolactone (PCL)

PCL is a thermoplastic polyester characterized by easy processability, biocompatibility, and good in vivo degradation rate, broadly used in tissue engineering applications [105]. However, by itself, it neither provides the cell adhesive domains nor factors to induce cell differentiation. Therefore, the incorporation of additives in PCL scaffolds is often proposed, such as other biopolymers or inorganic inclusions. Composite materials composed of PCL and hyaluronic acid (HA), a glycosaminoglycan widely distributed in the ECM, were shown to increase the differentiation potential. Jang et al. [106] have proposed hybrid PCL/HA microspheres that induce osteogenic differentiation of human periosteum-derived cells and significantly promote bone formation in vivo. In another study, the chondrogenic differentiation of hMSCs (human MSCs) was stimulated by electrospun nanofibers of acetylated HA/PCL composite [107]. Also, the addition of inorganic particles stimulated the differentiation of bone marrow-derived stromal cells to osteogenic lineage. The study on the composite of PCL and carbon nanotubes (CNT), fabricated by the solution evaporation approach, has proved the dependency of osteogenic differentiation on CNT concentration. Scaffolds with relatively lower CNT concentration (0.5%) were preferred over higher concentrations and had the osteoinductive potential [108]. The nanocomposite of PCL with magnesium (Mg) hydroxide nanoparticles also promoted osteogenic differentiation of hMSCs, with enhanced bone-specific matrix deposition [109]. In a study by Halabian et al. [110], it was found that by coating polyaniline-gelatin-PCL composite electrospun nanofiber with the addition of bioceramic nanoparticle (Zn_2_SiO_4_), the osteogenic differentiation of human-induced pluripotent stem cells (hiPSCs) was improved.

##### Topology

Due to its good processability, PCL, often with other polymeric additives, is used to produce a 3D electrospun scaffold characterized by high porosity, tunable mechanical properties, and proper biocompatibility. Electrospun fibrous scaffolds induced cell differentiation, facilitated by the scaffold’s specific typology (architecture) or stiffness.

While both PCL and PLA are linear aliphatic polyesters, they are different in terms of molecular structure. Thus, PCL is a more robust, hydrophobic, and crystalline polymer with slower degradation kinetics than PLA. Conversely, PLA is stiffer and tougher than PCL. When PLA and PCL are blended, the advantages of both polymers can be retained, while their drawbacks can partially be reversed [111]. Herrero-Herrero et al. [111] prepared PCL/PLA (polylactic acid) electrospun meshes that led to improved chondrogenic differentiation of ADSCs when compared to the pure PCL or pure PLA fibrous scaffold, even without the addition of a specific growth factor. Xu et al. [112] have shown that osteogenic differentiation is dependent on PCL/PLA weight ratio. PCL/PLA nanofiber blend of 20/80 weight ratio showed relatively higher stiffness when compared to PCL/PLA blend of 100/0 and 60/40 (Young’s modulus = 55; 8 and 45 kPa, respectively), favoring the osteogenic differentiation. Baudequin et al. [113] used PCL/PLA electrospun scaffolds to induce the osteogenic and tenogenic differentiation of the MSCs model in the absence of a specific differentiation medium. Whereas the native PLA could not initiate any differentiation, the scaffold composed of well-aligned pure PCL fibers (600–1000 nm) pushed stem cells towards bone differentiation, while the coaxial PCL/PLA blend (2000 nm fiber diameter) pushed stem cells towards tendon lineage. This study indicated the importance of the scaffold topology. In the study by Su et al. [114] it was also proved that ADSCs cultured on plasma-modified electrospun PCL fiber showed multidirectional or bi-directional growth patterns on random and aligned fiber patterns, respectively. An interesting study conducted by Ghobeira et al. [115] has demonstrated that the fibrous topography of electrospun PCL fiber can modulate the paracrine function of adipose-derived stem cells (ADSCs), consequently promoting wound healing in rat models.

##### Stiffness

To modulate the stiffness of fabricated scaffolds, a PCL mix with polytetrahydrofuran (PTHF) and collagen type 1 was also proposed. The obtained soft nanofiber electrospun scaffolds, with 4.3 MPa modulus, induced increased chondrogenic differentiation of hMSCs (in vitro studies) and cartilage regeneration (in vivo studies), and improved tissue regeneration by specifically blocking the NF-kappa B signaling pathway to reduce inflammation, when compared to the stiffer scaffold without collagen (Young’s modulus = 6.8 MPa) [116]. The addition of PLA was proposed by Yao et al. [117] to increase the mechanical stiffness and bioactivity of the rendered scaffolds, osteogenic differentiation in vitro, and bone formation in the in vivo model.

##### Biological Cues

Apart from material, stiffness and topological cues, the addition of biological factors could also induce stem cell differentiation. Olvera et al. [118] used electrospun PCL scaffolds to demonstrate the role of the growth factors incorporation and fiber alignment on the differentiation. Scaffolds composed of fibers with random and aligned orientation were seeded with bone marrow mesenchymal stem cells (BMSCs) and incubated with two growth factors, transforming growth factor β3 (TGF-β3) and connective tissue growth factor (CTGF) individually and sequentially, with distinct induction media. It was found that the combination of PCL fiber orientation with growth factor showed differentiation to a specific lineage. The aligned and random PCL fiber cultured with TGF-β3 showed chondrogenic and endochondrogenic differentiation, respectively; an aligned PLC fiber with CTGF and TGF-β3/CTGF demonstrated ligamentous and fibro-chondrogenic differentiation, respectively. The influence of PCL on cell differentiation is presented in Table 1.

#### 3.1.2. Polylactic Acid, L-lactic Acid, and lactic-co-Glycolic Acid (PLA, PLLA, and PLGA)

Polylactic acid (PLA) is a thermoplastic polyester formed by condensation of lactic acid formed by loss of water. Poly L-lactic acid (PLLA) and poly lactic-co-glycolic acid (PLGA) are the most common derivatives of PLA. Briefly, PLLA is an enantiomer of polyester PLA while PLGA is a co-polymer of glycolic acid and lactic acid. PLA and its derivatives have been extensively used in tissue engineering due to their tunability in mechanical properties, biocompatibility, ease of production, and recyclability [119].

Alike PCL, PLA has been employed in combination with various polymers, coatings, or additives to stimulate lineage-specific differentiation. The osteogenic differentiation of hMSCs using surface-modified 3D printed PLA was shown by Jaidev et al. [120]. The 3D printed PLA scaffold was surface functionalized with polyethyleneimine and citric acid conjugation, followed by immersion in simulated body fluid with calcium-deficient hydroxyapatite. The surface-modified scaffold, after culturing, depicted a relatively higher mineral deposition in comparison to the non-functionalized scaffold, indicating hMSCs osteogenesis of the system. In another study, it was demonstrated that the polydopamine and collagen type 1 coating of 3D printed PLA scaffolds improved the BMSCs metabolism and ECM deposition, consequently supporting osteogenic differentiation [121]. Osteogenic differentiation of ADSCs has been proven by using 3D printed PLA microstructure encapsulated within photo-curable gelatin hydrogel. The gelatin served as a source of cyclic RGD (arginine-glycine-aspartic acid) conjugated gold nanoparticles (RGNPs). Markedly, the incorporation of RGNPs has resulted in higher expression of the bone-specific gene and promoted osteogenesis of ADSCs [122].

2D scaffolds, obtained by Ojaghi et al. [123] and 3D electrospun scaffolds were cultured alike in media with differentiation growth factor and stimulated by glucose. However, only 3D PLLA/PVA system showed a relatively high expression level of islet genes and C-peptide and insulin release, proving the efficacy of the 3D PLLA/PVA system. In another study, curcumin-loaded PLLA/PHB (poly(3-hydroxybutyrate) electrospun fibrous scaffolds were shown to induce osteogenesis in ADSCs with higher expression of osteogenic markers in comparison to native mat devoid of curcumin [124]. The addition of pluronic, which improve the hydrophilic properties of polymers like PLLA, was studied by Birhanu et al. [125]. The work has proved that the pluronic blended PLLA electrospun fiber scaffolds not only provide an improved surface for adherence and proliferation of stem cells but also drive osteogenic differentiation of human ADSC cultured in an osteoinductive medium. It was also shown that osteogenic differentiation could be induced by electrospun composite scaffolds composed of PLLA mixed with oyster shell (with compositional and crystalline resemblance to human bone) [126], with functionalized octa calcium phosphate [127] or electrospun PLLA scaffold coated with bioactive glass-ceramic nanoparticle [128].

Rezaei et al. have proved that PLGA-PU electrospun nanofibrous scaffolds with poly-phosphate (Poly-P) introduced to the electrospun fiber during the preparation of the PLGA-PU blend), can enhance smooth muscle cell differentiation from hADSCs. The study has shown that Poly-P can trigger mTOR and Akt signaling pathways involved in SMC modulation [129].

In a recent study, the neural differentiation of MSCs in neural induction media was induced on PLGA-CNT microsphere containing alginate. The effect was assigned to CNT-facilitated stem cell adhesion, and alginate provided an optimal environment for the growth and differentiation of MSCs [130].

##### Topology

To study the effect of substrate chemistry and microstructure on modulating the signaling pathway and stem cell differentiation, PLLA-based substrate of different topography (flat and fibrous) and chemistry (pristine and aminated) was studied by Li et al. [131]. The authors have found that the synergic effect of fibrous and aminated PLLA could trigger a differential gene expression of BMSCs in comparison to a flat and pristine PLLA, differentiation of human MSCs. In another study, it was found that the electrospun PLLA nanofibrous scaffolds, with aligned topology, promoted the osteogenic differentiation of ADSCs via modulation of IncRNAs (long noncoding RNAs) and microRNA (miR-125b). The study concluded that nanotopographical cues could stimulate molecular mechanisms of osteogenic differentiation [132].

**Figure 2:**
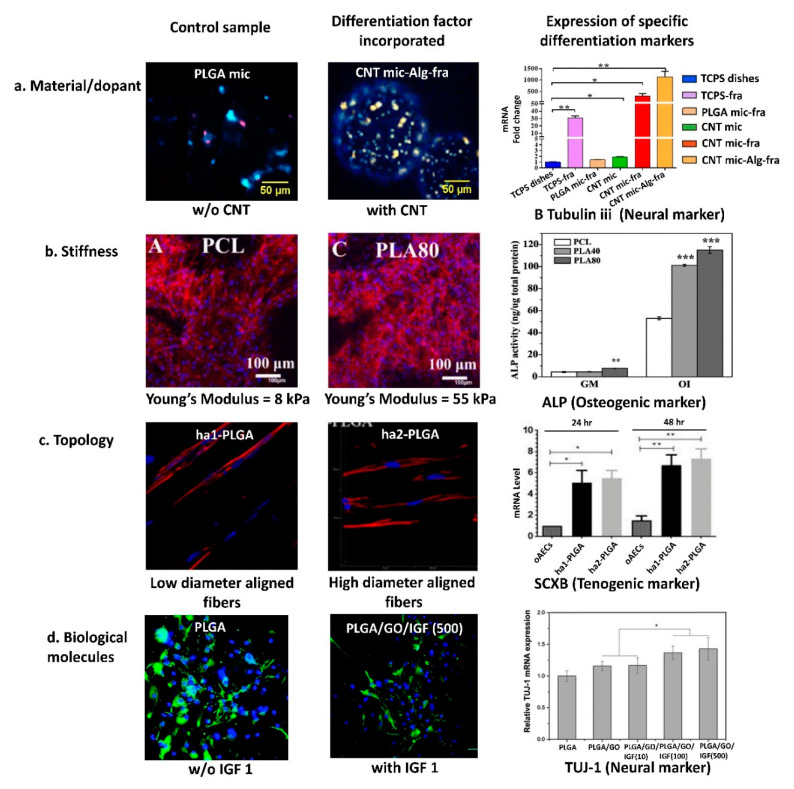
Factors inducing differentiation of stem cells in synthetic materials with selected examples. (**a**) Material or dopant chemistry. Alginate gel with carbon nanotube (CNT)–containing PLGA microsphere (CNT mic-Alg-fra) induced differentiation of neural stem cells (NSCs) in the presence of neural differentiation media. Left and middle column: Immunohistochemistry analysis after 8-day culture indicating the differentiation of NSCs can be improved by the presence of CNT in comparison to the control (PLGA mic: PLGA microsphere without differentiation factor). Red: B tubulin iii (neural marker); blue: DAPI staining of nuclei. Right column: Expression of β-tubulin iii measured for: control tissue culture plates without (TCPS dish) and with (TCPS-fra) differentiation media, PLGA microsphere with differentiation factors (PLGA mic-fra), PLGA-CNT microsphere without (CNT mic) and with (CNT mic-fra) differentiation media and, PLGA-CNT microsphere enclosed in alginate gel with differentiation factor (CNT mic-Alg-fra). CNT mic-Alg-fra revealed significantly higher expression of the differentiation marker. Adapted with permission from [130]. (**b**) Scaffold stiffness. 3D electrospun meshes with increased stiffness-induced osteogenic differentiation of human mesenchymal stem cells (hMSCs) in osteoinductive media. Left and middle column: Immunohistochemistry analysis of 16 h culture indicating that PLA 80 (20% PCL–80% PLA) electrospun meshes with relatively higher stiffness improved hMSCs differentiation in comparison to less stiff pure PCL meshes (PCL). Red: Texas Red-Phalloidin staining of F-actin; blue: DAPI staining of nuclei. Right column: Higher activity of alkaline phosphate was detected in stiffer samples (PLA 80) in comparison to less stiff samples PLA 40 (60% PCL–40% PLA) and control (PCL). Adapted with permission from [117]. (**c**) Scaffold topology. Electrospun PLGA nanofiber meshes composed of fibers with higher (2.5 µm) diameter (ha2-PLGA) improved tenogenic differentiation of amniotic epithelial stem cell (oAECs) in comparison to lower (1.27 µm) diameter PLGA (ha1-PLGA) electrospun meshes. Left and middle column: Immunohistochemistry analysis of oAECs in 2 days culture on ha2-PLGA and ha1-PLGA scaffolds showed tenocyte-like elongated morphology in the teno-inductive media. Red: Phallodin staining of actin; blue: DAPI staining of nuclei. Right column: ha2-PLGA demonstrated a higher expression of SCXB (tenogenic marker) in comparison to ha1-PLGA and control samples of oAECs cultured in Petri dishes. Adapted with permission from [135]. (**d**) Bioactive molecules. The addition of the IGF-1 growth factor into graphene oxide incorporated electrospun PLGA fiber (PLGA/GO/IGF-1) increased the differentiation of NSCs in neuro-inductive media. Left and middle column: Immunofluorescence staining of astrocyte marker (GFAP) in 7 days cell culture of NSCs showed that IGF-1 at a concentration of 500 ng/mL (PLGA/GO/IGF-1 (500)) improves the differentiation of NSCs in comparison to control (PLGA). Green: GFAP (astrocyte marker); blue: DAPI staining of nuclei. Right column: PLGA/GO/IGF-1 (500) demonstrated a higher expression of neuron-specific marker (TUJ-1) when compared to material containing 10 ng/mL and 100 ng/mL IGF-1 (PLGA/GO/IGF (10) and PLGA/GO/IGF (100), respectively and control (PLGA). *—*p* < 0.05; **—*p* < 0.01; ***—*p* < 0.001. Adapted with permission from [133].

An electrospun PLGA scaffold composed of aligned fibers with varying diameters revealed the influence of this parameter on the tenogenic differentiation of amniotic epithelial stem cells. The lower diameter PLGA fiber (1.27μm) was a better mimic of the tenogenic microenvironment and boosted tenogenic differentiation than the higher diameter fiber (2.5 µm) [133]. In another study, the aligned electrospun PLGA fibers revealed improved neural differentiation of mouse embryonic stem cells, in comparison to random ones, due to contact guidance with neurites on the extended fiber axis [134]. Figure 2 below provides examples of various factors inducing the differentiation of stem cells in synthetic materials.

##### Stiffness

The effect of PLLA-based electrospun scaffolds stiffness on cell differentiation was also shown by Mirzaei et al. [136] using an electrospun nanofibrous mesh composed of PLLA/polyethylene glycol (PEG) mix loaded with glucosamine. Chondrogenic differentiation was detected on the PLLA-PEG 20,000 scaffolds that are composed of high molecular weight PEG (20,000) and characterized by relatively higher stiffness, in comparison to the native PLLA fiber and PLLA-PEG 3000 scaffolds that are composed of low molecular weight PEG (3000) that revealed lower stiffness. In another study, the electrospun mesh was prepared of PLLA modified with PHBV (poly(3-hydroxybutyrate-co-3-hydroxy valerate) indicated osteogenic differentiation of mouse BMSCs under non-osteogenic conditions. The effects were assigned to an increase in material glass transition temperature and Young’s modulus of the scaffold due to the incorporation of PHBV [137].

The 3D porous composite scaffold of PLLA-PLGA obtained by salt leaching technique, with different elasticity (Young’s modulus range of 60–280 kPa), was obtained by changing the PLLA to PLGA ratio (100–25% PLLA) and revealed influence on myoblast differentiation. The results have shown that a compliant scaffold (modulus of 60 kPa) is insufficient to withstand cellular force, while firm scaffolds (with Young’s modulus in the range of 200–280 kPa) could not support the parallel alignment of myoblast. Thus, it was concluded that the optimal stiffness of the PLLA/PLGA scaffold has to be tailored to direct specific stages of myoblast differentiation and organization [138]. In another study, injectable micro-ribbon-shaped fibronectin-coated PLGA scaffolds with increased stiffness (value of 62–68 MPa) led to the osteogenic differentiation of hMSCs [139].

##### Biological Cues

The significance of biochemical cues like growth factors and protein is an inevitable factor that aids in stem cell differentiation. PLGA has been augmented by various growth factors to trigger differentiation to distinct cell lineage. In a study conducted by Wei et al., it was proved that soybean lectin-mediated PLGA microspheres with nanoporous topology had shown an improved osteogenic differentiation in the presence of bone morphogenic protein-2 (BMP2) [140]. Other studies performed using graphene oxide-incorporated electrospun PLGA nanofiber scaffolds incubated with IGF1 have illustrated that the combined effect of graphene oxide and IGF1 can induce neural stem differentiation [133]. The role of growth factor was also exemplified using a knitted PLGA-fibrin gel scaffold loaded with basic fibroblast growth factor (bFGF) and MSC, which demonstrated tenogenic differentiation of MSCs [141]. The influence of PLA and its derivatives on cell differentiation is presented in Table 2.

#### 3.1.3. Polyethylene Glycol and (Ethylene Glycol) Diacrylate (PEG and PEGDA)

PEG and its derivative (PEGDA) also have been widely used in stem cell differentiation applications. Nachlas et al. [142] investigated the intrinsic ability of PEGDA to initiate valve interstitial cell maturation. Human iPSC–derived mesenchymal stem cells (iMSCs) encapsulated in PEGDA hydrogel grafted with RGD adhesion peptide were differentiated into valve interstitial-like cells. Noh et al. [142] induced osteogenic differentiation of ADSCs via the incorporation of graphene oxide into PEGDA hydrogel. The authors concluded that graphene oxide could act as bio functionalizing moiety activating focal adhesion kinase signaling to improve cell adhesion, consequently promoting stem cell differentiation under osteoinductive conditions. In another study, it was observed that coating of electrospun polyethersulphone-PEG fibrous scaffold with bioceramic nanoparticle (Zn_2_SiO_4_) coating could enhance osteogenic differentiation of hMSCs over Zn_2_SiO_4_ non-coated scaffold [143].

The mechanical properties of PEG hydrogel scaffolds also have been explored for their stem cell differentiation properties. The influence of stress relaxation on MSCs differentiation was studied by Nam et al. [144] PEG with = short (2 kDa), medium (5 kDa) or long (20 kDa) chains at different degrees of substitution was grafted to the alginate. It was observed that the stress relaxation was decreased with increasing PEG concentration and increasing PEG chain length and was dependent on the total amount of PEG in the system. Increased PEG mass also led to increased creep, determined by the total mass amount of PEG. The study showed that faster relaxation of the gels promoted cell spreading, proliferation, and osteogenic differentiation. A polyamidoamine (PAMAM) dendrimer-PEG hydrogel system also revealed stiffness dependency on differentiation to specific cell lineage. The stiffness was varied by changing the concentration of the material. It was found that encapsulated MSCs showed osteogenic differentiation in relatively stiffer gel (5663 Pa) and adipogenic differentiation in relatively softer gel (77 Pa) when cultured in distinct induction media [145]. Table 3 summarizes the influence of polyethylene glycol and its derivative on cell differentiation.

### 3.2. Other Polymeric Biomaterials

#### 3.2.1. Polyurethane (PU)

Polyurethane is a family of synthetic biomaterials with exceptional flexibility and durability. Shahrousvand et al. [147] have demonstrated that 2D PU scaffolds with low stiffness (26 MPa) and high roughness, which in turn, dependent on the material concentration, can enhance osteogenic differentiation. Similarly, in another study using PU- poly(2-hydroxyethyl methacrylate)-cellulose nanowhisker scaffold prepared by solvent casting/particulate leaching method, the presence of carbon nanowhisker has proved to improve the mechanical properties that induce osteogenesis [148].

#### 3.2.2. Polyvinyl Alcohol (PVA)

Hazeri et al. [149] have employed PVA in combination with sulfated alginate to provide scaffolds inducing neural differentiation of MSCs. Electrospun nanofibers of PVA/sulfated alginate (30%) are an optimal substrate for neural differentiation, even in the absence of any growth factor. Their study has further paved the way for recent work on electrospun curcumin-incorporated chitosan/collagen/PVA nanofibrous scaffolds that have shown the SMC differentiation of iPSCs under culturing in differentiation media [150]. The study by Hou et al. [151] has demonstrated that the PVA microgel system loaded with BMP2, prepared by microfluidic technology, can stimulate the osteogenic differentiation of MSCs.

#### 3.2.3. Polyethylene Terephthalate (PET)

Polyethylene terephthalate (PET), a common thermoplastic polymer, was shown to promote osteogenic differentiation of BMSCs (in vitro) and osteointegration (in vivo) after integration with strontium-substituted hydroxyapatite [152]. The influence of other polymeric biomaterials on cell differentiation is summarized in Table 4.

### 3.3. Ceramics

Graphene-oxide reinforced ceramic nanofiber network was shown to induce neurogenic differentiation of hMSCs due to the topological and mechanical features of the scaffold. The graphene-augmented inorganic nanofiber (GAIN) with self-alignment and highly anisotropic nature and nanostructured topology, triggered Nestin (neuroepithelial stem cell protein) signal in the absence of specific differentiation media to induce neurogenesis [153].

Composite materials based on hydroxyapatite (both natural and synthetic) manufactured by sintering have been recently proposed as bone substitutes [154]. The hydroxyapatite served as a matrix and was doped with: (i) organic: multiwalled carbon nanotubes (MWCNT), fullerenes C60, (ii) inorganic: Cu nanowires. The selected samples exhibited bacteriostatic properties against Gram-positive reference bacterial strain S. epidermidis (ATCC 12228); however, the property was much less pronounced against Gram-negative reference strain *E. coli* (ATCC 25922). Both natural- and synthetic hydroxyapatite-based sinters, as well as their doped derivates, displayed good general compatibility, with the exception of Cu-nanowire doped derivates [154].

### 3.4. Metals

Metals like titanium and tantalum also have been explored for the stem cell differentiation potential and have induced osteogenic differentiation (Table 5) [155]. While those materials could be used alone, usually upon some surface modifications, a recent report [156] discusses their combined use (coating) with long-resorbable biomaterials based on polycaprolactone (PCL) without or with various dopants. The presented results show variable biological responses depending on the modifications to titanium plates and applied to PCL dopants.

## 4. Natural and Synthetic Biomaterials and Dopants Attenuating or Impairing Stem Cell Differentiation

Biomaterials for stem cells have emerged as a critical component in regenerative medicine since they can function as a biomimetic platform for relevant biological research [157]. Stem cells can self-renew and develop into one or more specialized cell types [158]. Stem cells are classified into embryonic stem cells and adult stem cells [159]. Incorporating stem cells into structured biomaterials improves the ability to restore and repair damaged tissues [40]. Therefore, combining stem cells with biomaterial scaffolds is a potential technique for creating tissues in vitro and in vivo [160].

The differentiation of stem cells can be regulated in the extracellular environment by using physical and chemical stimuli. Traditional cell culture methods based on soluble factors have limited effectiveness in controlling the stem cells’ fate [157]. Biomaterials, by mimicking the in vivo microenvironment, open up a novel route for influencing stem cell destiny through cell-matrix interactions. Biomaterial scaffolds can give cell attachment sites while still preserving the benefits of stem cells. Cell adhesion, cell transportation, cell differentiation, and matrix architecture can all be altered to control stem cell fate following rational designing [40]. 

### 4.1. Two-Dimensional (2D) Surfaces Versus Three-Dimensional (3D) Biomaterial Scaffolds

Traditionally, researchers used 2D surfaces for culture to control stem cell development. Recently, stem cells reside in the complex microenvironment or 3D biomaterial scaffolds, and their fate is regulated by various parameters owning to the extracellular matrix and surrounding cells. 2D cultures have more limited applications due to their limited dimensions, while 3D cell cultures have shown extraordinary promise in mimicking cell heterogeneity, spatial organization, biochemical composition, and mechanical properties of the main tissue [152]. 

### 4.2. Natural Versus Synthetic Biomaterials

A wide variety of natural and synthetic biomaterials have been tested as substrates for controlling stem cell differentiation. Natural biomaterials are highly biocompatible. However, synthetic biomaterials can be deliberately designed for a specific purpose [40,160]. Natural materials can signal to encapsulate cells through a variety of methods, including surface receptor interactions and degradation by cell-instructive enzymes. Although natural biomaterials have preferred biocompatibility and self-existing biosignals, their brittle mechanical strength and difficulty in their modification limit their applicability (compared to synthetic biomaterials). Synthetic biomaterials contain a wide range of characteristics, and processing synthetic materials into desired structures may be easier than with natural materials. Limitations of some synthetic materials include a limited repertoire of cellular interaction and toxicity unless they are modified by adhesion peptides [40]. Instructive biomaterials can be manufactured with greater control and repeatability than their natural equivalents by engineering biological activity into synthetic materials [157]. 

### 4.3. Microenvironment-Related Factors Affecting Stem Cells Fate

The use of external biophysical stimuli to regulate cell fate, such as adhesion, proliferation, migration, differentiation, and death, is a significant factor in modulating cell functions. Therefore, the microenvironment in which stem cells live determines their fate, and synthetic materials have been developed to mimic these regulatory processes for a variety of medical uses. In the microenvironment of stem cells, several factors affect their fate, which can be referred to as soluble factors, including growth factors or cytokines, nutrients, and bioactive molecules (ligands); cell−cell interactions; cell-biomaterial or biomacromolecule interactions; and physical factors, including the rigidity of the environment [161]. 

### 4.4. Parameters for Designing Biomaterials

Design parameters of materials (material architecture and mechanical properties) can be mentioned in engineering and materials mechanics, which provides the basis for receptor-ligand interactions and, therewith, can determine the fate of uncommitted stem cells [162]. Scaffolds, when properly configured, can directly govern cell signaling and stimulate lineage-specific differentiation of stem cells via chemical cues or cell-matrix interactions [40]. 

Biomaterials must be engineered to respond to the cell so that cells can detect and interact with them. Immobilization of cell-detecting ligands on biomaterials is a method to provide bioresponsive elements to materials. On the other hand, cell-adhesive ligands provide sites for the attachment of cells to the biomaterial. For example, one peptide sequence found in fibronectin and collagen is arginine-glycine-aspartate (Arg-Gly-Asp, RGD), which is responsible for cell adhesion to the extracellular matrix [163]. Therefore, the Arg-Gly-Asp peptide has become a real ligand for cell adhesion and chemical modification in biomaterials design [164].

Also, the biomaterials can be rationally designed by stimuli-responsive linkers and adhesive ligands [165]. This ON-OFF switch can change the access of adhesive ligands for stem cell binding and providing temporal control over stem cell adhesion [166]. For example, firstly, the surface of the glass platform was modified by Arg-Gly-Asp, followed by functionalizing with an elastase-sensitive dialanine linker and fluorenylmethyloxycarbonyl as a blocking group. These groups sterically prevent cells from interacting with the Arg-Gly-Asp ligands, which is called the “OFF” state. After applied stimuli, the removal of the fluorenylmethyloxycarbonyl blocking group by cleavage of the elastase-sensitive dialanine linker exposes Arg-Gly-Asp to the stem cells, resulting in stem cell adherence with Arg-Gly-Asp. This state represents the “ON” state. Therefore, the adhesion changes could further influence stem cell phenotype and control stem cell growth [167]. Another important factor involved in regulators of stem cell renewal and differentiation is soluble factors secreted by cells, such as growth factors and cytokines, because of their interactions with cells and the extracellular matrix [157,168]. The typical strategy for regulating stem cell phenotypes has been to stimulate stem cells using soluble substances. Growth factors, as soluble factors, can bind to receptors and activate cellular signal transduction pathways that promote cell growth and differentiation [169]. A summary of growth factors utilized in stem cell differentiation is included in Table 6.

Therefore, the slower release of growth factors encapsulated within biomaterials can regulate cell response and matrix formation [202]. Controlled growth factors release can be achieved by modifying the properties of biomaterials. For example, collagen/chitosan/silk fibroin scaffolds containing transforming growth factor β1 encapsulated in polylysine-heparin sodium nanoparticles could control the slow release of TGF-β1 and modulate the mouse mesenchymal stem cells (mBMSCs) to differentiate into chondrocytes and osteoblasts [203]. In addition, the immobilization of growth factor proteins on biomaterial surfaces can be a beneficial strategy for programmable manipulation over cell differentiation pathways. For example, the controlled immobilization and displacement of the fibroblast growth factor (FGF-2) and bone morphogenetic protein (BMP-2) were demonstrated on an advanced vapor-based coating of poly[(4-2-amide-2′-amine-dithiobisethyl-p-xylylene)-co-(pxylylene)]. The disulfide exchange mechanism of the advanced vapor-based coating enables the detachment and/or displacement of the previously installed growth factor protein, allowing for the reinstallation of a second growth factor protein. Cleavage of growth factor proteins can reduce or stop previously induced biological activity, whereas reinstallation of a different type of second-factor protein can re-start divergent differentiation activity [204].

### 4.5. The Influence of Properties of Extracellular Matrices on Cell Fate

In addition, the properties of extracellular matrices can influence cell fate and steer tissue growth (i.e., differentiation into specific lineages) [205]. Physical interactions between cells and the elasticity (or rigidity and stiffness) of the ECM in which they are cultivated, in particular, can impact stem cell destiny, despite the fact that stem cell fate has traditionally been assigned to genetic or molecular mediators. Many studies have recently realized that the flexibility of cell culture substrates defines hMSC lineage commitment. When cultivated on biomaterials with similar elasticity to those tissues, stem cells differentiate more efficiently into specific tissue lineages. The elasticity of cell culture substrates, particularly in 2D culture, can clearly influence cell morphology, cell phenotype, and focal adhesions [205]. The mechanical sensing of substrates by stem cells is thought to be caused by integrin-mediated focal adhesion signaling [206]. Integrins are receptors that mediate cell-ECM attachment in cell culture substrates or tissues. They are made up of obligate heterodimers with two distinct chains of subunits. Integrins contribute to cell-matrix signaling by activating intracellular tyrosine kinase and phosphatase signaling, which results in downstream biochemical signals that regulate gene expression and stem cell fate [207]. Some materials have stimuli-switchable properties. Stimulus-responsive hydrogels, especially hydrogels with stimuli-tunable mechanical properties, provide an important platform for the development of novel materials with specific applications [208]. A summary of the main strategies to modulate hydrogel stiffness is included in Table 7.

Various additives differently affect hydrogels’ mechanical properties. For example, the polyacrylamide-based hydrogel containing photo-switchable cross-linkers was able to modulate bone-marrow-derived mesenchymal stem cells’ behavior through alteration of substrate mechanics in response to stimulation that was otherwise “invisible” to the cells. This hydrogel can reversibly alter its stiffness upon irradiation with the appropriate wavelength of light. In other words, the synthesized hydrogel is shown that near-UV irradiation leads to softening of the gel, whereas visible blue light leads to stiffening, which alters cell morphology [220].

### 4.6. Assessment of Time-Dependent Responses of Stem Cells 

Controlling the display of bioactive ligands on biomaterial scaffolds is very desirable for the regulation and investigation of time-dependent responses of stem cells. For example, a magnetically responsive platform including a soft hydrogel substrate conjugated with Arg-Gly-Asp tripeptide-bearing magnetic nanoparticle (RGD-MNP) was able to regulate the adhesion, migration, and differentiation of human mesenchymal stem cells. According to magnetical-responsive hydrogel, the upward magnetic attraction promotes the presentation of the RGD-MNP ligands (EXPOSED state) on the soft hydrogel matrix to generate a cell−adhesive surface that enhances the mechanosensing of human mesenchymal stem cells, while downward magnetic attraction conceals the presentation of the RGD-MNP ligands (HIDDEN state) and inhibit cell mechanosensing. Therefore, differentiation/dedifferentiation of human mesenchymal stem cells is induced by cyclic switching between Exposed and Hidden conditions, respectively [221].

Controlling the nanoscale presentation of bioactive ligands remotely, non-invasively, and reversibly is extremely desirable for temporally regulating cellular functions in vivo. Therefore, designing materials with sticky ligands that can be regulated remotely is an appealing strategy for non-invasive and temporal control of cell adhesion in vivo [222]. Magnetic fields have good penetration of living tissues with minimum cytotoxicity, making them appropriate for remotely regulating the mobility of magnetic nanoparticles and clinical applications [223,224,225]. For example, temporal switching of the ligand oscillations between high- and low-frequency modes reversibly regulated human mesenchymal stem cell adhesion and differentiation [226]. Remotely inducing slow or fast ligand oscillations by adjusting the frequency of an oscillating magnetic field significantly promoted or inhibited integrin−ligand binding and the substrate adhesion of stem cells, respectively, both in vitro and in vivo, therewith significantly regulating the differentiation of the stem cells.

Also, the heterodimeric magnetic nanoswitch was able to remotely control and regulate in vivo adhesion and differentiation of stem cells on the heterodimer coupled substrate by reversibly manipulating Arg-Gly-Asp-bearing gold nanoparticle caging and uncaging. Magnetically controlled movement of magnetic nanocage as a nanoswitch relative to Au nanoparticles under applied an external magnetic field allowed reversible uncaging and caging of Arg-Gly-Asp that regulated physical accessibility of Arg-Gly-Asp for integrin binding. Therefore, reversible Arg-Gly-Asp caging can temporally control stem cell adhesion and differentiation [222].

## 5. Conclusions and Future Perspectives

Cells are typically generated from either donor tissue, which is often in short supply, or stem/progenitor cells. The high proliferative capability and pluripotency, or the ability to develop into cells of numerous lineages, of stem cells make them particularly suitable for usage. Although there are ethical problems with using human embryonic stem cells, the use of induced pluripotent stem cells, adult stem cells, and stem cells obtained from placental and umbilical sources have largely supplanted embryonic stem cells as viable sources. The cellular microenvironment, which permits cells to behave as they do in native tissue, is one of the key aspects that must be considered for an optimal outcome in tissue engineering. This can be accomplished by employing suitable materials with the necessary mechanical and chemical qualities to mimic in vivo situations. 

Currently, Section 351-approved devices employ autologous cells, which require invasive biopsies and lengthy culture durations, or allogeneic differentiated cells, which raise safety issues [227]. It is crucial for scalable translation to identify an ideal source of cells that do not cause immunological rejection. Many of these cell sources are now being studied as cell treatments. Autologous bone marrow or adipose-derived MSCs with multilineage potential have been shown to be safe and effective in the treatment of a wide range of disorders and organs [228]. Most crucially, Yamanaka’s discovery of induced pluripotency in 2007 paved the way for a new generation of patient-specific cells [229,230]. The capacity to create functional differentiated cells of every tissue type from patient-derived fibroblasts has enormous tissue engineering applications [231]. Despite the fact that these cells necessitate sophisticated processes that cause side effects, his work encouraged changes for clinical accessibility and safety, such as the use of direct reprogramming and nonviral vectors [232]. Recently, considerable attention has been directed toward in situ direct reprogramming, which has promising applications in cell/material therapy [233,234]. Furthermore, several investigations have been conducted in order to discover an allogeneic stem cell source. Placental-derived stem cells, for example, have been demonstrated to function similarly to MSCs without eliciting an immunological response [228]. Other approaches include using viral vectors to eliminate stem cell human leukocyte antigen expression, resulting in an “off the shelf” donor cell that may be used on any patient without eliciting an immunological response [235].

Cell scaffolds could serve for cell adhesion and migration; retention of biochemical factors and their presentation; porous microenvironment for adequate diffusion of cells, nutrients, expressed products, and waste; and mechanical strength purposes. A wide variety of natural and synthetic biomaterials have been tested as substrates for controlling stem cell differentiation. Natural biomaterials are highly biocompatible. However, synthetic biomaterials can be deliberately designed for a specific purpose. Although natural biomaterials are being widely used, inconsistent purity arising from lot-to-lot variability and difficulty in sterilization and purification is usually the main limitations of natural biomaterials. Synthetic biomaterials, non-biological in origin, marked their position among the materials for tissue engineering and regenerative medicine, as they are relatively easy to manufacture at a large scale, with high flexibility and control over their composition, microstructure, degradation rate, mechanical properties, and possible functionalization. Natural materials can signal to encapsulate cells through a variety of methods, including surface receptor interactions and degradation by cell-instructive enzymes. Although natural biomaterials have preferred biocompatibility and self-existing biosignals, their brittle mechanical strength and difficulty in their modification limit their applicability. Synthetic biomaterials contain a wide range of characteristics, and processing synthetic materials into desired structures may be easier than with natural materials. Limitations of some synthetic materials include a limited repertoire of cellular interaction and toxicity unless they are modified by adhesion peptides. Instructive biomaterials can be manufactured with greater control and repeatability than their natural equivalents by engineering biological activity into synthetic materials. 

To facilitate particular tissue growth, the ideal scaffold should contain biological and mechanical components [236]. Various 3D platforms stand out for their capacity to promote cell survival, tissue development, and integration after implantation. Decellularized tissues, whether intact or processed from the allogeneic or xenogeneic origin, allow for the use of native ECM, which impacts cellular activity positively [237,238]. By eliminating biological components, the matrix may be transplanted into any patient without rejection, increasing its applicability [239,240]. Various efforts have been made to develop optimal decellularization procedures as well as storage settings. Many tissue types’ decellularized matrices have been clinically investigated and are commercially accessible for a variety of reasons [239,240]. Furthermore, decellularized entire organs preserve circulatory networks and can be reseeded with autologous cells for tissue maturation prior to transplantation [241,242,243].

Hydrogels can also be utilized to construct complicated tissues. Hydrogels with customized qualities have been created using both natural and synthetic materials or their combination. Gelatin methacryloyl, for example, has tunable mechanical properties, functionalization, cell encapsulation, drug elution, degradation, and smart, responsive behavior, allowing for adaptation to a variety of organ systems [244,245]. Tuning the compositions of material properties and growth factors in acellular and cellular approaches to mimic the native environment has been found to be effective in promoting healing [246]. Recent research into the production of tailored hydrogels from patient biopsies seeded with autologous cells has potential uses for a wide range of organ types with a low risk of immunological rejection [247].

Physical and chemical stimuli can be used to control stem cell development in the extracellular environment. Traditional cell culture methods based on soluble factors are ineffective in controlling the fate of stem cells. Biomaterials, by simulating the in vivo microenvironment, provide a new avenue for influencing stem cell fate via cell-matrix interactions. Biomaterial scaffolds can provide cell attachment sites while retaining stem cell advantages. Following rational design, cell adhesion, cell transportation, cell differentiation, and matrix architecture may all be adjusted to regulate stem cell destiny. External biophysical stimuli used to influence cell fate, such as adhesion, proliferation, migration, differentiation, and death, are important factors in modifying cell activities. As a result, stem cells’ destiny is determined by the microenvironment in which they exist, and synthetic materials have been designed to imitate these regulatory mechanisms for a range of medicinal applications. Several factors influence stem cell fate in their microenvironment, including soluble factors such as growth factors or cytokines, nutrients, and bioactive molecules (ligands); cell-cell interactions; cell biomaterial or biomacromolecule interactions; and physical factors such as environment rigidity. 

Many tissue techniques have been demonstrated to be viable on the bench, but generating these tissues at economically relevant scales remains a hurdle. Cell generation and 3D printing of bigger tissues with viable processes are continuing projects. Many companies, including Organovo, Allevi, and CELLINK, are now focusing on the latter [227]. Furthermore, new efforts such as the Advanced Regenerative Manufacturing Institute are funding studies to uncover crucial components required to scale-up viable technologies to reach patients. The commercialization of developed technologies and therapeutics is critical for the broad application of tissue engineering.

Some natural materials have received FDA approval for tissue engineering, and several authorized devices are based on these materials [248]. Collagens [249,250,251] and glycosaminoglycans, such as hyaluronic acid [251,252], chondroitin sulfate [249,251,253], and chitosan [254], are common materials in this class. The origin of these natural materials determines their biocompatibility and function. Collagens are often collected from bovine or porcine tissues, whereas glycosaminoglycans in cartilage are produced from animal sources or bacterial production. While these materials may elicit an enhanced immune response in some cases, these naturally synthesized and purified materials have been found to be safe on the whole [255].

A variety of FDA-approved synthetic polymers have also been used as structural components in tissue engineering scaffolds. PEG [256], PLGA [257], PCL, and ultra-high molecular weight polyethylene are examples (UHMWPE) [258]. These materials have found widespread clinical use in adhesives, sutures, medical devices, and joint replacement. Although the chemistry of these materials is not biomimetic, matching mechanical qualities and water content can be adequate to elicit tissue-appropriate cell responses. This is thought to occur because when a biomaterial is implanted, native proteins adsorb to the surface, modulating cell-material interactions. To match the mechanical characteristics of native tissues, the mechanical properties of both natural and synthetic polymers and materials must be modified. This needs a fundamental grasp of polymer theory. Certain polymers, such as rigid rod peptides with hydrogen bonding capability, are inherently stiffer than a flexible, hydrophilic PEG chain with a random coil structure. Furthermore, by employing the same material system but increasing the molecular weight of the polymers, integrating shorter cross-linkers, or introducing more crosslinking junctions, the modulus and ultimate strength of the material may be considerably increased [259].

## Figures and Tables

**Table 1:** PCL and cell differentiation.

Differentiation Factor		Type of Scaffold	Differentiation	Ref.
Material/dopant	HA	PCL/HA microsphere prepared by spray precipitation	Osteogenic	[106]
	Acetylated HA	PLC/HA electrospun nanofibrous scaffold	Chondrogenic	[107]
	CNT	Cylindrical scaffold of PCL/CNT prepared by solution evaporation technique	Osteogenic	[108]
	Magnesium	PCL/Mg nano composite film	Osteogenic	[109]
	Bioceramic (Zn_2_SiO_4_)	Polyaniline-gelatin-PCL composite electrospun nanofiber with ceramic nanoparticle (Zn_2_SiO_4_) coating	Osteogenic	[110]
Topology/stiffness	Relative increase	PCL/PLA electrospun mesh	Osteogenic	[117]
	PCL/PLA random meshes of 1.8 µm diameter	PCL/PLA electrospun mesh	Chondrogenic	[111]
	Young’s Modulus of 55 kPa	PCL/PLA electrospun mesh	Osteogenic	[112]
	PCL aligned fiber of 1000 nm diameter Coaxial PCL/PLA blend of 2000 nm diameter	Electrospun scaffold of coaxial PCL/PLA fibers	Osteogenic Tenogenic	[113]
	Soft scaffold of PTHF/PCL/collagen type 1 (Modulus of 4.3 MPa)	PTHF/PCL/collagen type 1 electrospun nanofiber	Chondrogenic	[116]
Biological molecules/topology	TGF-β3/random fibers TGF-β3/aligned fibers TGF-β/CTGF/aligned fibers CTGF/aligned fibers	PCL electrospun sheets ca. 300 µm thick	Endochondral Chondrogenic Fibrochondrogenic Ligamentous	[118]

**Table 2:** Influence of PLA and its derivatives on cell differentiation.

Differentiation Factor		Type of Scaffold	Differentiation	Ref.
Material/dopant	Polyethyleneimine, citric acid and hydroxyapatite	3D printed PLA with the functionalized surface	Osteogenic	[120]
	PDL/collagen type 1	3D printed PLA with PDL and collagen type 1 coating	Osteogenic	[121]
	Gelatin/RGNPs	3D printed PLA embedded in Gelatin/RGNPs	Osteogenic	[122]
	PVA addition	Electrospun nano fibrous scaffold	Pancreatic cells	[123]
	PHB (stiffness) curcumin	Electrospun nano fibrous scaffold	Osteogenic	[124]
	Pluronic	Electrospun nano fibrous scaffold	Osteogenic	[125]
	Oyster shell	Electrospun nano fibrous scaffold	Osteogenic	[126]
	Octa calcium phosphate	Electrospun nano fibrous scaffold	Osteogenic	[127]
	Bioactive glass ceramic nanoparticle	Electrospun nano fibrous scaffold coated with ceramic nanoparticle	Osteogenic	[128]
	Poly-P solution	Electrospun nano fibrous scaffold	Myogenic	[129]
	CNT	PLGA-CNT microspheres with alginate hydrogel	Neurogenic	[130]
Stiffness	Relatively high stiffness	Electrospun nano fibrous scaffold	Chondrogenic	[136]
	Relatively high Young’s modulus value	Electrospun nano fibrous scaffold	Osteogenic	[137]
	Young’s modulus > 200 kPa	3D porous composite bulk scaffold prepared by salt leaching	Myogenic	[138]
	Young’s modulus 62–68 MPa	Injectable micro-ribbon shaped fibronectin coated PLGA	Osteogenic	[139]
Topology	Aligned fibers	Electrospun fibrous scaffold	Osteogenic	[132]
	Lower fiber diameter (1.27 μm)	Electrospun nano fibrous scaffold	Tenogenic	[133]
	Aligned fibres	Electrospun nano fibrous scaffold	Neurogenic	[134]
Biological molecules	BMP2	Soybean lectin mediated PLGA microspheres	Osteogenic	[141]
	GO/IGF1	Electrospun nano fibrous scaffold	Neuronal	[133]
	Fibrin/bFGF	Knitted gel scaffold	Tenogenic	[141]

**Table 3:** Influence of polyethylene glycol and its derivative on cell differentiation.

Differentiation Factor		Type of Scaffold	Differentiation	Ref.
Material/dopant	PEGDA/RGD	Bulk hydrogel	Heart valve interstitial cells	[146]
	GO	Bulk hydrogel	Osteogenic	[142]
	Zn_2_SiO_4_	Electrospun nano fibrous scaffold with Zn_2_SiO_4_ coating	Osteogenic	[143]
Stiffness	Relatively increased relaxation time and creep	Bulk hydrogel	Osteogenic	[144]
	Hard gel (5.7 kPa) Soft gel (77 Pa)	Bulk hydrogel	Osteogenic Adipogenic	[145]

**Table 4:** Influence of other polymeric biomaterials on cell differentiation.

Differentiation Factor		Type of Scaffold	Differentiation	Ref.
Material/dopant	Low stiffness (Young’s modulus = 26 MPa) and high roughness	Bulk scaffold	Osteogenic	[147]
	Cellulose nanowhisker	Porous bulk scaffold	Osteogenic	[148]
	PVA/sulphated alginate	Electrospun nanofibrous scaffold	Neurogenic	[149]
	Chitosan/collagen/PVA nanofiber with Curcumin	Electrospun nanofibrous scaffold	Myogenic	[150]
	PET/SR-HA	Bulk material	Osteogenic	[152]
Biological Molecules	BMP-2	Microgel system	Osteogenic	[151]

**Table 5:** Influence of ceramics and metals on cell differentiation.

Differentiation Factor		Type of Scaffold	Differentiation	Ref.
Material/dopant	Ceramic/GO	Nanofibrous scaffold	Neurogenic	[153]
	Titanium	Bulk material	Osteogenic	[155]

**Table 6:** Effects of various growth factors on stem cell differentiation.

Growth Factor	Observed Effects	Ref.
BMP subfamily	Enhanced proliferation, and differentiation to osteogenic and chondrogenic phenotype	[170,171,172,173,174,175]
EGF	Enhanced proliferation, viability, and migration	[176,177,178]
FGF	Enhanced proliferation and differentiation to chondrogenic phenotype	[179,180,181]
HGF	Enhanced proliferation and viability	[182]
IGF-1	Enhance proliferation and viability, influencing apoptosis, differentiation to neural phenotype	[183,184,185,186,187,188,189]
Insulin	Differentiation to adipogenic phenotype	[190]
PDGF	Enhanced proliferation and viability, differentiation to the pulmonary, neural, dermal, skeletal, gastrointestinal, vessel, and hematopoietic cell types	[191,192,193]
TGF-β family	Enhanced proliferation, differentiation to oligodendric and chondrogenic phenotype	[194,195,196,197,198]
VEGF	Enhanced proliferation and viability, differentiation to hematopoietic, osteogenic, and chondrogenic phenotypes	[184,185,187,189]
Wnt family	Enhanced proliferation and differentiation	[199,200,201]

**Table 7:** Different approaches are utilized for the modulation of hydrogels’ mechanical properties.

Materials	Concentration	Preparation Method	Range of Elastic Modulus	Cells	Ref.
Alginate hydrogelsGelatin	0.8, 1.3, 1.8, and 2.3 wt% 4.1 wt%	CaCl_2_ cross-linking	1.4−14,2 kPa	^a^ hMSCs	[209]
Alginate hydrogelsGelatin	1, 3, 5, 7, and 9 wt% 2, 4, 6, 8, and 10 wt%	CaCl_2_ cross-linking	29.8−48 kPa	^b^ MSCs	[210]
Silk fibroin	1.5−4 wt%	high-pressure CO_2_	6−64 kPa	MSCs	[211]
Silk fibroin Collagen	various ratio starting from silk fibroin (70 mg/mL) collagen (7.8 mg/mL)	Gelatin	9.93−31.16 kPa	^c^ BMSCs	[212]
Silk fibroin Collagen	0.175−2.45 wt% 0.5−3.5 mg/mL	sonication and gelation	0.05−20.4 kPa	hMSCs	[213]
Silk fibroin	2 wt%	freeze-drying	3−58.4 kPa	BMSCs differentiation in endothelial cells	[214]
Silk fibroin nanofiber	5 wt%	concentrating silk fibroin nanofiber solution and salt leaching	2−18 kPa	BMSCs myogenic differentiation	[215]
Silk fibroin Silk fibroin nanofiber	various ratio starting from 6% (silk fibroin) and 2% (silk fibroin nanofiber)	^d^ HRP cross-linking	9−60 kPa	BMSCs differentiation in different lineages	[215]
Silk fibroin nanofiber	1, 2, and 4 wt%	HRP-cross-linking	0.6−160 kPa	BMSCs	[216]
Gelatin methacrylamide	5, 10, and 15 wt%	photo-crosslinking	1.7−16.4 kPa	dental stem cells	[217]
Gelatin methacrylamide	7.5, 10, and 15 wt%	photo-crosslinking	25.59−41.78 kPa	BMSCs differentiation into endothelial cells	[218]
Gelatin methacrylamide	10 wt%	photo-crosslinking	3.5−13.1 kPa	human adipose- derived stem cells	[219]

^a^ Human Mesenchymal Stem Cells (hMSCs); ^b^ Mesenchymal Stem Cells (MSCs); ^c^ Bone marrow mesenchymal stem cells (BMSCs); ^d^ horseradish peroxidase (HRP)

## Data Availability

Not applicable.

## Abbreviations

Arg	arginine
Asp	aspartic acid
Au	gold
bFGF	basic Fibroblast Growth Factor
BMP2	Bone Morphogenic Protein-2
BMSCs	Bone marrow Mesenchymal Stem Cell
CA	Chitosan Alginate
CNT	Carbon NanoTubes
CTGF	Connective Tissue Growth Factor
dECM	decellularized Extracellular Matrix
ECM	Extracellular Matrix
EGF	Epidermal Growth Factor
FGF	Fibroblast Growth Factor
GAIN	Graphene Augmented Inorganic Nanofiber
gelMA	gelatin Methacrylate
Gly	Glycine
GO	Graphene Oxide
hADSCs	human Adipose-Derived Stem Cells
HGF	Hepatocyte Growth Factor
hiPSCs	human induced Pluripotent Stem Cells
hMSCs	human Mesenchymal Stem Cells
HRP	HorseRadish Peroxidase
IGF1	Insulin like Growth Factor-1
IncRNAs	long noncoding RNAs
MAPK/ERK	Mitogen-Activated Protein Kinases/Extracellular signal-Regulated Kinases
MCWCNT	MultiWalled Carbon NanoTubes
Mg	Magnesium
MSCs	Mesenchymal Stem Cells
mTOR	mammalian Target Of Rapamycin
Nestin	neuroepithelial stem cell protein
PAMAM	PolyAMidoAMine
PCL	PolyCaproLactone
PDGF	Platelet-Derived Growth Factor
PEC	PolyElectrolyte Complex
PEG	PolyEthylene Glycol
PEGDA	Poly(Ethylene Glycol)DiAcrylate
PET	PolyEthylene Terephthalate
PHB	Poly3-HydroxyButyrate
PHBV	Poly(3-HydroxyButyrate-co-3-hydroxyValerate)
PLGA	PolyLactic-co-Glycolic Acid
PLLA	PolyL-Lactic Acid
Poly-P	Poly-Phospate
PTHF	PolyTetraHydroFuran
PVA	PolyVinyl Alcohol
RGD	aRginine-Glycine-aspartic aciD
RGD-MNP	Arg-Gly-Asp tripeptide-bearing magnetic nanoparticle
RGNPs	RGD conjugated Gold Nanoparticles
SMC	Smooth Muscle Cell
TGF-β3	Transforming Growth Factor β3
VEGF	Vascular Endothelial Growth Factor
